# Differentiation and Molecular Properties of Mesenchymal Stem Cells Derived from Murine Induced Pluripotent Stem Cells Derived on Gelatin or Collagen

**DOI:** 10.1155/2016/9013089

**Published:** 2016-08-25

**Authors:** Chizuka Obara, Kazuya Takizawa, Kenichi Tomiyama, Masaharu Hazawa, Ai Saotome-Nakamura, Takaya Gotoh, Takeshi Yasuda, Katsushi Tajima

**Affiliations:** Research Center for Radiation Emergency Medicine, National Institute of Radiological Sciences, 4-9-1 Anagawa, Inage-ku, Chiba-shi, Chiba 263-8555, Japan

## Abstract

The generation of induced-pluripotential stem cells- (iPSCs-) derived mesenchymal stem cells (iMSCs) is an attractive and promising approach for preparing large, uniform batches of applicable MSCs that can serve as an alternative cell source of primary MSCs. Appropriate culture surfaces may influence their growth and differentiation potentials during iMSC derivation. The present study compared molecular properties and differentiation potential of derived mouse iPS-MSCs by deriving on gelatin or collagen-coated surfaces. The cells were derived by a one-step method and expressed CD73 and CD90, but CD105 was downregulated in iMSCs cultured only on gelatin-coated plates with increasing numbers of passages. A pairwise scatter analysis revealed similar expression of MSC-specific genes in iMSCs derived on gelatin and on collagen surfaces as well as in primary mouse bone marrow MSCs. Deriving iMSCs on gelatin and collagen dictated their osteogenic and adipose differentiation potentials, respectively. Derived iMSCs on gelatin upregulated* Bmp2* and* Lif* prior to induction of osteogenic or adipose differentiation, while PPAR*γ* was upregulated by deriving on collagen. Our results suggest that extracellular matrix components such as gelatin biases generated iMSC differentiation potential towards adipose or bone tissue in their derivation process via up- or downregulation of these master genes.

## 1. Introduction

Mesenchymal stem cell- (MSC-) mediated cytotherapy has attracted increasing interest owing to its safety and effectiveness in a number of auto-, allo-, and xenogeneic animal models [1, 2]. MSCs can be easily harvested from various adult human tissues and rapidly expanded* in vitro*, although this may limit their properties such as plasticity over the long term [3]. In addition, starting tissues such as bone marrow and adipose as well as culture conditions affect MSC properties and preparation, making it difficult to compare results from different MSC studies [4]. Induced pluripotent stem cells (iPSCs) are one of the most appropriate basic cell sources to not only generate standardized cell lines but also progenitor cells [5]. Therefore, iPSCs-derived mesenchymal stem cells (iMSCs) are considered as a promising alternative cell source of primary MSCs [6]. Indeed, iMSCs proliferate extensively* in vitro*, possibly resolving the challenge of generating large batches of identical primary MSCs [7].

Extracellular components in the stem cell niche define the stem cell function and specification by generating signals for survival, proliferation, and differentiation; however, the underlying mechanisms are only partially understood. Therefore, it is crucial to investigate how these components affect their properties [8, 9]. A previous report found that gelatin-coating on plates increased proliferation in bone marrow-derived MSCs (BMMSCs) as compared to cells cultured on uncoated plates [10]. Several reports have shown that iMSC characteristics such as differentiation potentials* in vitro* and* in vivo* are similar to those of primary progenitor cells [11], but it is not known whether extracellular components such as gelatin and collagen significantly affect iMSC characteristics in its derivation process. Although several studies have successfully reported the derivation of human iMSCs from iPSCs [3, 12–14], a simple and efficient method for inducing mouse iMSCs has not yet been established [15, 16]. However, the safety of further clinical applications must be evaluated in appropriate animal models including genetically modified mouse, which requires the establishment of simple and effective derivation method for mouse iMSCs in addition to human cells.

In the present study, we designed culture plates-coated with gelatin or collagen and derived iMSCs from mouse iPSCs using these plates. After their derivation, we investigated whether the derived iMSC differentiation potential is dictated towards bone or adipose tissue using differentiation assay with uncoated plates.

## 2. Materials and Methods

### 2.1. Mouse Induced Pluripotent Stem Cells (iPSCs) Culture

Three mouse iPS cell (iPSC) lines (2A-4F-60, 2A-4F-100, and 2A-4F-136) were kindly gifted by Dr. Araki and Dr. Abe [17]. Cells were maintained on mouse embryonic fibroblast (MEF) in basal medium consisting of Dulbecco's modified Eagle's medium (DMEM) with 15% knockout serum replacement, 2 mM nonessential amino acids (NEAA), 2 mM L-glutamine (all from Invitrogen, Carlsbad, CA, USA), 0.1 mM 2-mercaptoethanol, and leukemia inhibitory factor (LIF; 500 U/mL, ESGRO, Merck Millipore, Billerica, MA, USA).

### 2.2. MSC Isolation and Culture

Prospectively isolated BMMSCs over mouse bone marrow were prepared as previously described [18, 19]. BMMSCs were isolated from three C57Bl/6 mice (5 to 9 weeks old), which were purchased from Japan SLC Inc. (Shizuoka, Japan). In brief, femur and tibiae were removed, cleaned, and cut into fine pieces. The bone fragments were incubated for 1 h at 37°C in DMEM in the presence of 10 mM HEPES, 0.2% collagenase (Wako Pure Chemical Industries, Osaka, Japan), and 5 U/mL DNase I (Takara Bio, Shiga, Japan). After digestion, they were filtered through a 70-*μ*m cell strainer (BD Falcon, Franklin Lakes, NJ) and centrifuged at 1200 rpm (270 g) for 10 min in a Kubota rotor RS-720 (Kubota, Tokyo, Japan). The cell pellet was resuspended in 1 mL of sterile distilled water for 6 s to disrupt the red blood cells, and then 1 mL of 2x Dulbecco's phosphate-buffered saline (D-PBS; Nissui Pharmaceutical, Tokyo, Japan) containing 4% fetal bovine serum (FBS; HyClone, GE Healthcare Life Sciences, UT, USA) was added. The cell suspension was filtered through a cell strainer and centrifuged at 270 g for 5 min. The pellet was resuspended in fresh Hank's Balanced Salt Solution containing 10 mM HEPES, 2% heat-inactivated fetal bovine serum (FBS) (HyClone, GE Healthcare Life Sciences, Logan, UT, USA), and 1% penicillin/streptomycin (HBSS+) and then stained with allophycocyanin-conjugated PDGFR*α* fluorescein isothiocyanate-conjugated Sca-1, phycoerythrin-conjugated CD45, and phycoerythrin-conjugated TER-119 (all from eBiosciences, San Diego, CA, USA) for 30 min at 4°C. The cells were washed with HBSS+, stained with 7-aminoactinomycin D (Beckman Coulter, Fullerton, CA, USA) to exclude dead cells, and sorted using the FACSAria cell sorter (Becton Dickinson; now BD Biosciences, San Jose, CA, USA). The fraction containing MSCs (Sca-1^+^/PDGFR*α* ^+^/CD45^−^/Ter119^−^) was sorted by adequate cell gating.

The isolated MSCs were cultured in DMEM supplemented with 10% FBS and antibiotics. When the cells reached ~70% confluence, they were passaged using 0.25% trypsin/EDTA (Thermo Fisher Scientific). Expanded MSCs were passaged 4 (P4) to 7 times and used for all experiments.

### 2.3. Derivation of Induced Mesenchymal Stem Cells (iMSCs) from iPSCs Using Gelatin or Collagen-Coated Plates

**Figure 1:**
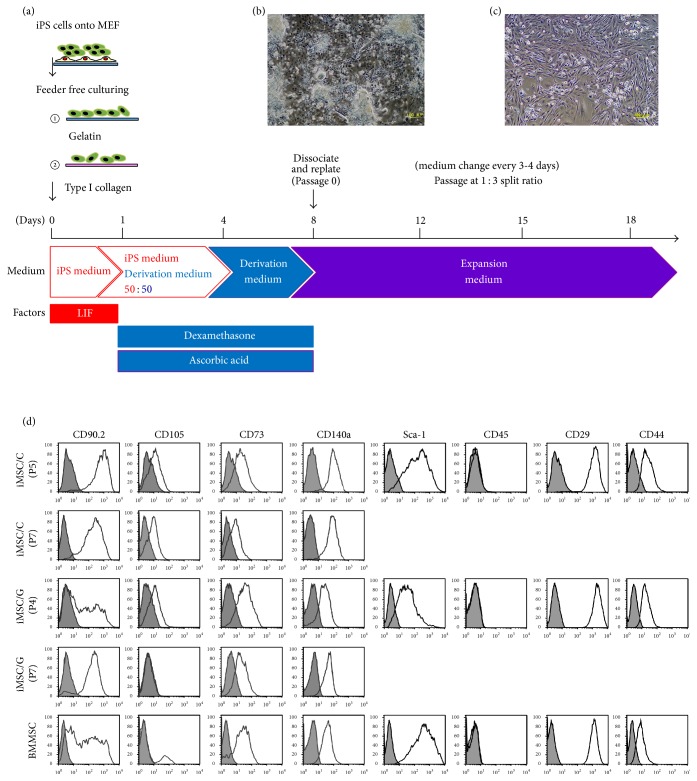
Derivation of mouse iPSC-derived mesenchymal stem cells (iMSC). (a) Protocol for derivation of iMSCs. iPSCs were maintained on mouse embryonic fibroblast (MEF) in iPS medium with leukemia inhibitory factor (LIF) and harvested and dissociated into single cells that were seeded into gelatin ①- or collagen ②-coated plates. After 1 day, the iPS medium was supplemented with an equal volume of the derivation medium with dexamethasone and ascorbic acid, and after 2 days, the medium was replaced with derivation medium. After 8 days, cells (passage zero; P0) were harvested and replaced in new gelatin- or collagen-coated plates in expansion medium. (b) Adherent spindle-shaped fibroblast-like cells at P0 appeared on 8-derivation day. (c) Homogenous fibroblast-like cells at P3. (d) Characterization of iMSCs on gelatin (iMSCs/G) and on collagen (iMSCs/C) at P4 to P7. Bone marrow (BM) MSC at P5 was provided by cell sorting from mouse bone marrow. The gray histogram represents the negative control staining with fluorescence-conjugated isotype IgG and white overlay represents antigen at CD90.2, CD105, CD73, CD29, CD44, CD45, Sca-1, and CD140*α*.

We applied a modified one-step method for the derivation of mouse iMSCs based on a previously described method for human iMSCs (Figure 1) [3]. Briefly, mouse iPSCs were harvested with 0.05% trypsin/EDTA and dissociated into single cells that were seeded in the gelatin or collagen-coated plates in 10 cm diameter (Iwaki; AGC TECHNO GLASS Co., Ltd., Shizuoka, Japan), at a density of 15,000/cm^2^ in the basal medium. MEF cells were excluded from iPSCs treated with removal microbeads conjugated with anti-feeder antibody (Miltenyi Biotec, Tokyo, Japan) (Catalog #130-095-531) in every derivation experiments. In brief, mixture solution of iPSCs and MEF cells were adjusted to more than 1 × 10^7^ cells in 80 *μ*L and incubated with 20 *μ*L Feeder Removal Microbeads at 4°C for 20 min. After incubation, the solution which was diluted by 500 *μ*L with 5% FBS in DMEM and was collected after being thrown into LS Columns (Miltenyi Biotec) (Catalog #130-042-401) to remove MEF cells. The collagen-coated plates (Iwaki) (Catalog #4020-010) were made of dried pepsin-soluble type I collagen extracted from pig tendons. The gelatin-coated plates (Iwaki) (Catalog #4020-020) were made of dried gelatin extracted from pig skins. After 24 h, the basal medium was supplemented with an equal volume of the derivation medium comprising basal alpha-minimum essential medium (*α*-MEM) (Invitrogen), 10% FBS (Vitromex, San Antonio, TX, USA), 100 nM dexamethasone, and 50 *μ*M magnesium L-ascorbic acid phosphate (all from Sigma-Aldrich, St Louis, MO, USA). After 2 days, the medium was completely replaced with the derivation medium without ROCK inhibitor, which was used in derivation of human iMSCs [3], and 8 days later, cells were harvested, thoroughly dissociated, and designated as passage zero (0). Single cells were cultured in new collagen- or gelatin-coated plates in alpha-MEM expansion medium containing 1% penicillin/streptomycin, 10% FBS, NEAA, and L-glutamine. The expansion medium was replenished every 3-4 days. iMSC like cells cultured on gelatin (iMSCs/G) or on collagen (iMSCs/C) were passaged when they reached 70% subconfluency at a ratio of 1 : 3 as completely single cell suspensions. Derivation iMSCs P4 to P7 times were used for all experiments. Derivation experiment of every iPSC clones was performed at five times.

### 2.4. RNA Preparation and Semiquantitative Reverse Transcription Polymerase Chain Reaction (RT-PCR)

RNA was extracted from P5 iPSCs, BMMSCs, iMSCs/G, iMSCs/C, and mouse MSCs (Invitrogen, Carlsbad, CA, USA) using an RNeasy Mini Kit (Qiagen, Valencia, CA, USA) according to the manufacturer's instructions with on-column DNase I digestion. Mouse MSCs were cultured in DMEM supplemented with 10% FBS and antibiotics under 5% CO_2_ at 37°C. When the cells reached ~70% confluence, they were passaged by using 0.25% trypsin/EDTA. Expanded MSCs at P4 to P7 times were used for all experiments. RT was carried out using the PrimeScript II First Strand cDNA Synthesis Kit (Takara Bio, Otsu, Japan). PCR amplification was performed in a PCR Thermal Cycler (Dice, Takara) using an Ex Taq (Takara) (Catalog #6210A), with the standard cycle condition. Germ-layer associated primers* Nanog, Oct4, *and* SOX2* for undifferentiated cells;* Nestin, Otx2, TP63/TP73L, SOX-2, *and* SOX-1* for ectodermal lineage;* AFP, GATA-4, PDX-1/IPF1, SOX17, *and* HNF-3b/FoxA2* for endodermal lineage;* Brachury* for mesodermal lineage were purchased from Mouse Pluripotent Stem Cell Assessment Primer Pair Panel Kit (R&D systems) (Catalog #SC015), and other primers (*PDGFR-α, PDGFR-β, ACTIN, *and* SOX1*) were prepared as previously described (Supplementary Table  1 in Supplementary Material available online at http://dx.doi.org/10.1155/2016/9013089) [20]. Total RNA (500 ng) was used as a template for cDNA synthesis with the RT^2^ First Strand (Qiagen) kit (Catalog #330401). The product was assessed by 1.5% agarose gel electrophoresis with ethidium bromide staining.

### 2.5. Microarray Analysis

P5 iPSCs, BMMSCs (P5), iMSCs/G (5-6P), and iMSCs/C (5-6P) were analyzed with a mouse mesenchymal stem cell RT^2^ Profiler PCR Array (Qiagen) (Catalog #PAMM-082Z). Real-time PCR was carried out on an ABI Prism 7500 system format C using a RT^2^ SYBR Green ROX*™* qPCR Mastermix (Qiagen) (Catalog #330522) under standard cycling conditions. Data were analyzed using ΔΔC_t_ method for 96-well format. The value of each sample was normalized to the expression level of the* GAPDH* housekeeping gene in the same sample. Relative mRNA expression level was expressed as fold-increases of the target genes to* GAPDH* mRNA level. A clustergram was generated by hierarchical clustering of genes and samples were displayed in a heat map, with dendrograms indicating coregulated genes across groups or individual samples. A scatter plot was used to compare the normalized expression of every gene in the array between the two selected groups by plotting them against one another. Every cell lines composed of three samples were analyzed at three times.

### 2.6. Flow Cytometric Analysis

Surface markers for mouse MSCs were quantified by flow cytometry using antibodies against CD11b, CD29 (encoded by* Itgb1* gene), CD44, CD73, CD90.2 (*Thy1*), CD105 (*Eng*) (from eBiosciences, San Diego, CA, USA), CD31, CD34, CD45, Sca-1, and PDGFR-*α* (Biolegend, San Diego, CA, USA) antibodies. Nonspecific fluorescence was determined with isotype-matched antibodies (BD Biosciences, Franklin Lakes, NJ, USA) (Supplementary Table  2). BMMSCs, iMSCs/G, iMSCs/C, and iPSCs (1 × 10^5^ cells) were collected by trypsinization and washed once with DMEM supplemented with 5% FBS. The sample was resuspended in 100 *μ*L of 5% FBS/PBS containing each of monoclonal antibodies and then incubated for 30 min at 4°C. The nonspecific binding of APC, FITC, and PE conjugates was determined using isotype antibodies (all from eBiosciences). The cells were then washed twice and resuspended in 400 *μ*L of 5% FBS/PBS. Cells were sorted on a FACSCalibur flow cytometer (BD Biosciences) and results were analyzed with Flow Jo software (Tree Star, Ashland, OR, USA). Flow cytometry analyses were repeated at least three times.

### 2.7. Differentiation Assay

We evaluated the potential of P5 iMSCs/G or iMSCs/C derived from each of iPSC lines to differentiate into adipocytes, osteocytes, and chondrocytes. These differentiations were performed using a human MSC differentiation kit (Lonza, Walkersville, MD, USA). Adipose differentiation was performed on normal coated plates and osteoblast differentiation on collagen-coated plates. Positive areas in Alizarin red for osteogenesis and Oil Red-O dye for adipogenesis were examined under higher magnification (4 or 10x) and quantitated using Image J. The positive density was expressed as the mean positive area per high-power field. A total of 4 high-power fields were randomly examined and counted from each treatment group (*n* = 3/group). The statistical significance of differences was determined using Student's *t*-test for two group comparisons or one-way analysis of variance for multiple comparisons, followed by Tukey's test. Differences with a value of *P* < 0.05 were considered significant. Differentiation assay for iMSC lines was repeated at three times.

## 3. Results

### 3.1. Derivation of Mouse iMSCs from iPSCs

To obtain the mouse MSC-like cells, we employed the modified version of the one-step derivation method used to obtain human MSC-like cells from iPSCs [3] (Figure 1(a)). With this method, adherent spindle-shaped fibroblast-like cells appeared in the derivation medium on 8-derivation day that continued to proliferate in the expansion medium (Figure 1(b)). After more than three passages, homogenous fibroblast-like derivation cells were obtained on both gelatin- and collagen-coated plates, which exhibited similar numbers of cells and doubling times (Figure 1(c)). Removal of MEF cells from iPSCs was completed using microbeads (Supplementary Figure  1).

### 3.2. Characterization of Surface Antigens in Fibroblast-Like Adherent Cells Derived from Mouse iPSCs

To characterize adherent fibroblast-like derivation cells, the expression of putative MSC markers was assessed at several time points. About 95% of derivation cells on collagen- or gelatin-coated plates were positive for putative MSC markers including Sca-1, CD29 (*Itgb1*), CD44, and CD73, but negative for CD11b, CD31, CD45, and CD117, independent of passage number (Figure 1(d)). In contrast, derivation cells heterogeneously expressed CD90 (*Thy1*) and PDGFR-*α* at P0 but gradually upregulated their markers dependent on passage numbers (Figure 1(d)). Expression of CD105 (*Eng*) was downregulated with increasing numbers of passages in derivation cells on gelatin, but not on collagen (Figure 1(d)). This heterogeneous expression of CD90 and CD105 was also detected in BMMSCs at P1 (Figure 1(d)). Our prospectively isolated BMMSCs using FACS analysis were negative for CD45. Collectively, the expression profiles in derivation cells on gelatin- or collagen-coated plates were similar to the general MSC profile [21]. Previous studies showed that immunophenotypic profiles of MSCs vary by species, cell source, and passage number [22–24].

### 3.3. Effects of Gelatin or Collagen on Germ-Layer Associated Genes Expression in iMSCs

**Figure 2:**
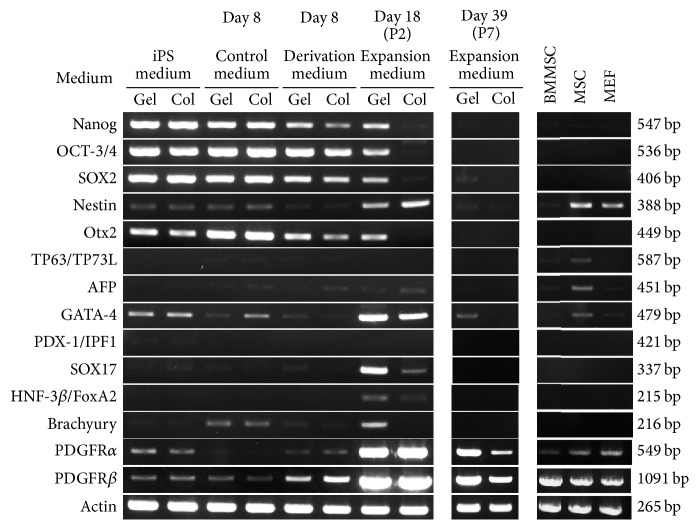
Effects of gelatin or collagen on germ-layer associated genes expression in iMSCs. Time-course (day 8 to day 39) genes expression in each cell with semiquantitative reverse transcription polymerase chain reaction. iPS medium; iPSCs on gelatin (Gel) or collagen (Col), control medium; iPSCs in 8 day-derivation without dexamethasone and ascorbic acid, derivation medium; iPSCs in 8 day-derivation, expansion medium; iMSCs at passages 2 (P2) and P7 in expansion medium, BMMSCs; bone marrow MSC at P5, MSC; MSCs from Invitrogen, and mouse embryonic fibroblast (MEF).

Stemness genes including* Nanog*,* Oct4*, and* Sox2* were expressed in iPSCs. Following derivation, their expression was gradually reduced and finally disappeared in iMSCs/C, although* Oct4* and* Sox2* transcripts persisted in iMSCs/G until 18-derivation day (Figure 2). Expression of Otx2 as an ectodermal lineage marker was also detected in iMSCs/G, but not iMSCs/C after derivation.* Nestin* (*Nes*) and GATA-4 levels were upregulated in both iMSCs after derivation, while SOX17 was markedly upregulated in iMSCs/G as compared to iMSCs/C.* Brachyury, PDGFR-α*, and* PDGFR-β* as a mesodermal lineage gene were upregulated in iMSCs/C and iMSCs/G following derivation. BMMSCs expressed* PDGFR-α* and* PDGFR-β* but had a little* Nestin, TP63/TP73L*, and* AFP* expression. These findings suggest that germ-layer associated gene expression profiles are similar in iMSCs/G and C, although stemness associated gene expression tended to persist.

### 3.4. Mesenchymal Stem Cell Associated Gene Expression in Mouse iPSCs, BMMSCs, iMSCs/G, and iMSCs/C

**Figure 3:**
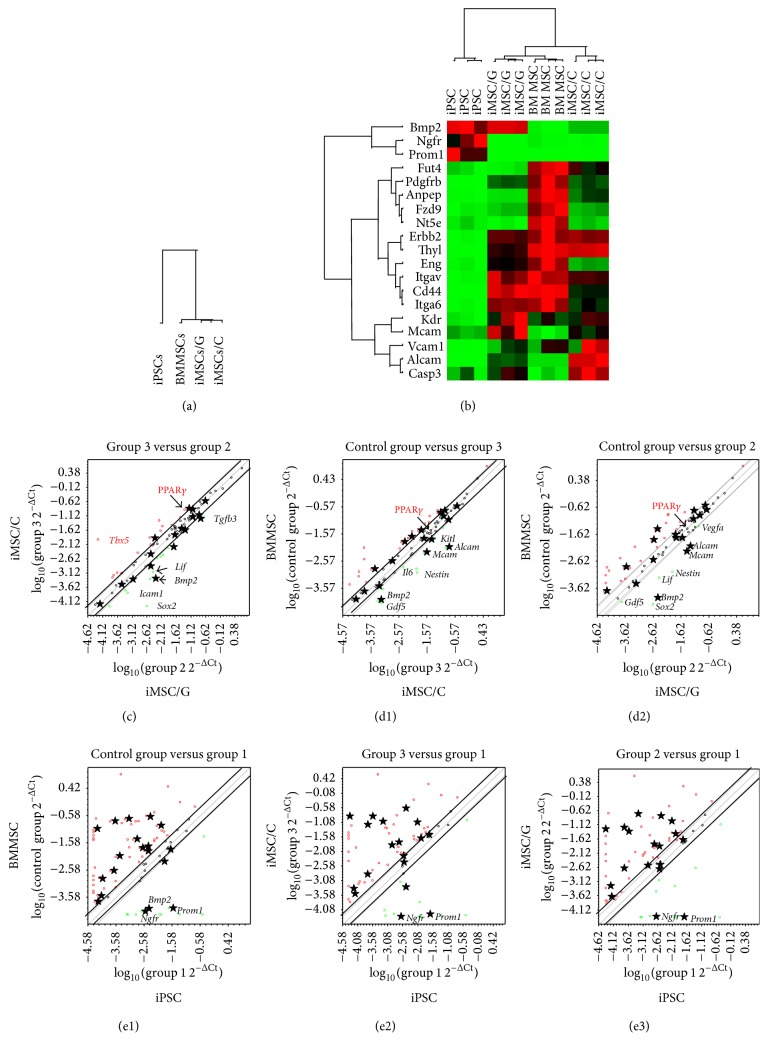
Microarray analysis of iMSCs/G, iMSCs/C, BMMSCs, and iPSCs. (a) Hierarchical cluster analysis of iMSCs/G, iMSCs/C, BMMSCs, and iPSCs by selecting 50 genes with upregulation by 2-fold in BMMSCs relative to iPSCs. (b) Hierarchical clustering analysis of MSC-specific genes of iMSCs/G, iMSCs/C, BMMSCs, and iPSCs. (c-e3) Pairwise scatter plots comparisons of the gene expression profiles of iMSC/C versus iMSC/G (c), BMMSC versus iMSC/C (d1)/iMSC/G (d2), BMMSC versus iPSC (e1), iMSC/C/iPSC (e2), and iMSC/G/iPSC (e3). Diagonal lines indicate the boundaries of twofold changes in gene expression. Gene expression levels are shown on log_10_ scale. MSC-specific genes: stars (★).

To investigate whether there is a molecular analogy among 4 cell groups as iPSCs, BMMSCs (P5), iMSCs/G (P5), and iMSCs/C (P5), we compared their gene expression profiles using a PCR array (Supplementary Figure  2). We performed a clustering analysis by selecting 50 genes that were upregulated by 2-fold in BMMSCs relative to iPSCs (Supplementary Figure  3). The 50 genes clustering analysis divided the cells into three clusters, iPSCs, BMMSCs, and iMSCs/G/C with a stronger correlation between iMSCs/G/C and BMMSCs as opposed to iPSCs (Figure 3(a)). We further examined 19 of 50 MSC-specific upregulated genes and found that BMMSCs and iMSCs/G and iMSCs/C were correlated by MSC-specific gene clustering (Figure 3(b)). A subclustering analysis based on six stemness genes upregulated* Lif* in iMSCs/G, but not in iMSCs/C. We added pairwise analyses (>2-fold), excluding genes with low expression selected by combining cell groups. The pairwise analysis between iMSCs/G and iMSCs/C revealed upregulation of* Sox2*,* Bmp2*, and* Lif* but downregulation of* Tbx5*, PPAR*γ*, and* Sox9* in iMSCs/G (Figure 3(c)). The scatter plots of twofold upregulated MSC-specific gene expressions (14 genes) demonstrated a resemble pattern (stars) between BMMSCs and iMSCs, except for common upregulation of* Bmp2*,* Mcam* (CD146), and* Alcam* (CD166) in iMSCs (Figures 3(d1) and 3(d2)). Alcam encodes CD166, which is a member of the immunoglobulin superfamily proteins.* Mcam* functions as a receptor for laminin *α*4, a matrix molecule. MSC-specific genes were generally upregulated in BMMSCs, iMSCs/G, and iMSCs/C compared with iPSCs except for* Ngfr* and* Prom1* (CD133) according to each pairwise analysis (Figures 3(e1)–3(e3)).* Ngfr* is a member of the tumor necrosis factor receptor superfamily, and* Prom1* encodes CD133 which is a member of pentaspan transmembrane glycoproteins [25]. Collectively, these results indicate that this one-step derivation method induced molecular similarities in terms of MSC-specific gene expression between BMMSCs and iMSCs/G or iMSCs/C.

### 3.5. Derivation of iMSCs on Gelatin-Coated Plates Enhances Osteoblast Differentiation Potential

**Figure 4:**
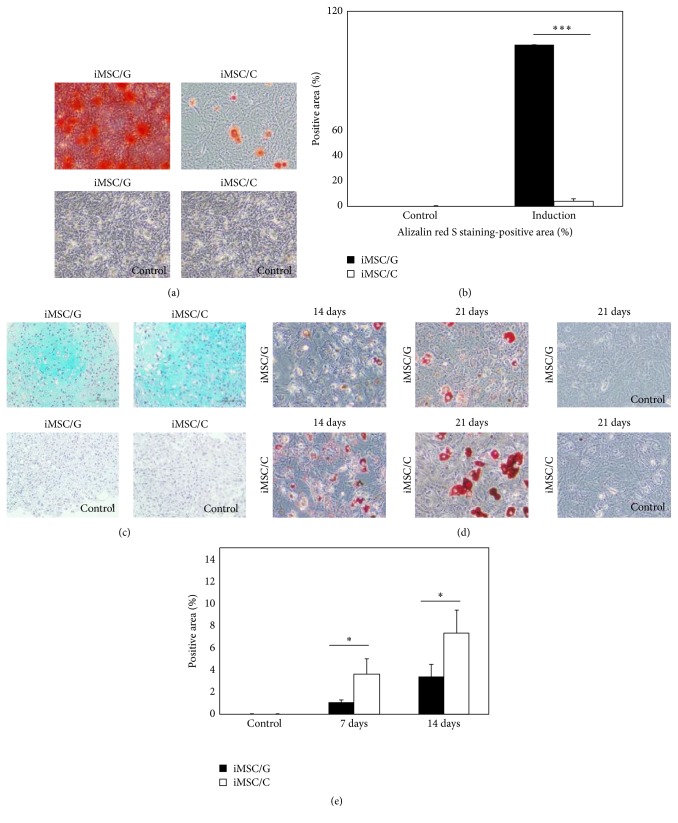
Osteogenic and adipose differentiation of iMSC/G and C derived from an iPSC clone (2A-4F-60). (a) iMSCs cultured in medium containing osteogenic differentiation factors (induction) or absence (control) and stained with Alizarin red at 28 days. (b) Relative amount of Alizarin red-positive areas. ^*∗∗∗*^ *p* value < 0.001. (c) Chondrocyte differentiation of iMSCs by micromass culture. (d) iMSCs cultured in differentiation medium (induction) or absence (control) and stained Oil Red-O at 14 and 21 days. (e) Relative amount of Oil Red-O-positive areas. ^*∗*^ *p* value < 0.05.

We assessed the osteogenic differentiation property of iMSCs/G and iMSCs/C. iMSCs were maintained at 70%–80% confluency on gelatin- or collagen-coated plates in osteogenic differentiation medium for 28 days, with medium replacement every 3-4 days. The majority of iMSCs/G demonstrated positive staining with Alizarin red and much amounts of its positive deposits, whereas a small fraction of iMSCs/C showed positive staining with a few amounts of deposits with quantification analysis (Figures 4(a) and 4(b)) (Supplementary Figure  4). There were no Alizarin red-positive cells on either substrate without induction of osteogenic differentiation. These findings at 28-osteoblast induction day suggested that iMSCs/G differentiate into osteoblasts more efficiently than iMSCs/C, which was further supported by the upregulation of* Bmp2* (6.50-fold) and* Lif* (4.77-fold) in iMSCs/G in pairwise scatter plots, prior to osteogenic induction. We performed the induction of chondrocyte differentiation of iMSCs by micromass culture method. The number of Alcian Blue positive cells was similar in both iMSCs on 24-differentiation day (Figure 4(c)), and the clustergram of chondrogenesis-associated gene expressions revealed molecular similarity between iMSCs/G and iMSCs/C.

### 3.6. Derivation of iMSCs on Collagen-Coated Plates Promoted Adipose Differentiation

Adipogenic differentiation of both iMSCs was examined on 7- and 21-differentiation day. The number of Oil Red-O-positive droplets was much higher in iMSCs/C as compared to iMSCs/G (Figures 4(d) and 4(e)) (Supplementary Figure  4). This indicates the propensity for adipogenesis in iMSCs/C. PPAR*γ* was upregulated by 2.18-fold in iMSCs/C according to pairwise analysis, prior to adipose induction.

## 4. Discussion

In this study, differentiation assay using uncoated plates revealed that derived mouse iPS-iMSCs on gelatin and collagen coating efficiently differentiated into osteoblast and adipose lineages, respectively. A previous study showed that rat BMMSCs showed increased proliferation and differentiation into a neurogenic lineage when grown on gelatin-coated plates as compared to noncoated plates, whereas adipose and osteogenic differentiation potentials were unaffected by gelatin [10]. In other studies using human or mouse derived iMSCs, osteogenic or chondrogenic differentiation potential was induced by gelatin or collagen coating, while they did not directly compare iMSC differentiation potential by deriving on the two types of substrates [3, 12, 15, 26]. The present study investigated for the first time how gelation or collagen coating affects the differentiation potential of derived iMSCs in culture.

In our pairwise scatter plot analysis, iMSCs/G upregulated* Bmp2* and* Lif* prior to induction of osteogenic or adipose differentiation, whereas iMSCs/C upregulated PPAR*γ*. MSCs can differentiate into bone and adipose tissue under the control of* Lif* and PPAR*γ* [27].* Lif* is a member of the interleukin-6 family of gp130 cytokines which stimulates osteoblast differentiation and reduces adipogenesis in bone marrow stromal cells [28, 29]. PPAR*γ* acts as a dominant negative regulator of osteoblast differentiation in BMMSCs [30] and inhibits* Lif*-induced proliferation of mouse embryonic stem cells [31]. However, the mutual interaction between* Lif* and PPAR*γ* in iMSCs has not been addressed and elucidated. Our present results suggest that, during iMSCs derivation from iPSCs, extracellular matrix composition biases its differentiation potential towards adipose or osteoblast lineages via up- or downregulation of these master genes. Gelatin which is composed of collagen-peptides can form three-dimensional complex structures, possibly inducing differences in germ-layer associated potentials of iPSCs. This hypothesis is compatible with previous reports [32–34]. However, three-dimensional relevance of gelatin remains unresolved in this study.

The cluster analysis of MSC-specific genes demonstrated that iMSCs/G or iMSCs/C were more closely related to BMMSCs than to iPSCs. This finding was confirmed by the pairwise scatter plot analysis which revealed a molecular similarity between BMMSCs and iMSCs/G or iMSCs/C, although they differed in terms of expression of* Alcam* and* Mcam* involved in cell adhesion molecules. We cultured BMMSCs on noncoated plates, which may explain differences in gene expressions between BMMSCs and iMSCs. A recent study showed that the gene expression profile of human iMSCs grown on gelatin-coated plates was similar to that of human BMMSCs by hierarchical cluster and MSC-specific gene heat-map analyses [12]. Nonetheless, our findings indicate that one-step iMSC derivation method can generate mouse iMSCs that have molecular characteristics compatible with those of primary mouse BMMSCs. The pairwise scatter plots demonstrated that the patterns of up- and/or downregulation of MSC-specific genes were similar between iPSCs and iMSCs as well as BMMSCs, suggesting the possibility that the process of* in vitro* iMSC derivation from iPSCs may reflect the generation process of BMMSCs* in vivo*.

As expected, we found that the expression of stemness related genes was gradually downregulated during the derivation of iMSCs from iPSCs. In addition, the expression stemness and germ-layer-specific genes persisted in iMSCs/G as compared to iMSCs/C. In contrast, germ-layer associated genes such as* Nestin, GATA4, SOX17, Brachury, PDGFRα*, and* PDGFRβ* were transiently upregulated in iMSCs at P2, but these genes were downregulated with additional passages, with the exception of* GATA4, PDGFRα*, and* PDGFRβ*. This is consistent with the gene expression profiles of human iMSCs [12, 13].

Previous studies have reported that expression levels of CD105 as an affinity coreceptor for transforming growth factor- (TGF-) *β*1 and TGF-*β*3 gene are correlated with MSC properties and are changed during* in vitro* expansion [14, 24, 35, 36]. In the present study, iMSCs/G gradually decreased CD105 expression over many passages and differentiated into osteoblasts more efficiently than iMSCs/C. This finding is in accordance with a previous study demonstrating that CD105 depletion enhanced human adipose-derived stromal cell osteogenesis via reduction of* TGF-β1* [24]. In this study, we observed upregulation of* TGF-β3* (3.01 fold), but not* TGF-β1* in iMSC/G.

## 5. Conclusion

We demonstrated the molecular properties of mouse iMSCs/G or iMSCs/C derived from iPSCs by a simple one-step derivation method. Deriving on gelatin enhanced the osteogenic differentiation potential of derived iMSCs via upregulation of* Lif* and* Bmp2*, whereas their adipogenesis was enhanced via upregulation of PPAR*γ* by deriving on collagen. These findings indicate that extracellular matrix components such as gelatin and collagen are critical regulators of the iMSC differentiation potential. The mouse iMSCs established by this method can be used as a tool for precisely analyzing anti-inflammatory or immunoregulatory roles of MSCs in a congenic mouse models, in addition to serving as an alternative cell source of primary MSCs and an off-the-shelf product.

## Supplementary Material

Supplementary Table 1 showed primers for PDGFRα, PDGFRβ, actin, and SOX1. Supplementary Table 2 showed all antibodies in this study. Supplementary Figure 1 showed removal of MEF cells using feeder removal microbeads and LC column. Supplementary Figure 2 showed microarray analysis of 78 gene expression profile. Supplementary Figure 3 showed clustering analysis by selecting 50 genes that were upregulated by two-fold in BMMSCs relative to iPSCs. Supplementary Figure 4 showed osteogenic and adipogenic differentiation of iMSC/G an C derived from 2A-4F-100 and 2A-4F-136 iPSC cell lines.
