# Supplementary material for: Differentiation and Molecular Properties of Mesenchymal Stem Cells Derived from Murine Induced Pluripotent Stem Cells Derived on Gelatin or Collagen
Source: Stem Cells Int. 2016 Aug 25;2016:9013089. doi: 10.1155/2016/9013089 (PMC5014975; doi:10.1155/2016/9013089)
Supplement: Supplementary file 1 — Supplementary Table 1 showed primers for PDGFRα, PDGFRβ, actin, and SOX1. Supplementary Table 2 showed all antibodies in this study. Supplementary Figure 1 showed removal of MEF cells using feeder removal microbeads and LC column. Supplementary Figure 2 showed microarray analysis of 78 gene expression profile. Supplementary Figure 3 showed clustering analysis by selecting 50 genes that were upregulated by two-fold in BMMSCs relative to iPSCs. Supplementary Figure 4 showed osteogenic and adipogenic differentiation of iMSC/G an C derived from 2A-4F-100 and 2A-4F-136 iPSC cell lines. [file 9013089.f1.pdf]

Supplementary Table 1

GENE	Forward	Reverse
PDGFR-α	5'-AATCCTGCAGACGAGAGCAC-3'	5'-GCCACCAAGGGAAAAGATTT-3'
PDGFR-β	5'-GTCTGGTCTTTTGGGATCCT-3'	5'-AAGGCTGGTTACAGTTTGGC-3'
β actin	5'-CCTAAGGCCAACCGTGAAAAG-3'	5'-TCTTCATGGTGCTAGGAGCCA-3'
SOX1	5'-CAATCTTGCATCCCGGTTC-3'	5'-ACCCAGGTCTTATCCCATCC-3'

Supplementary Table 1. Polymerase chain reaction primers for PDGFRα, PDGFR β, actin, and SOX1.

Supplementary Table. 2

Antibody	Conjugate	Clone	Catalog No.	Supplier
Anti-mouse CD11b	FITC	M1/70	11-0112	eBioscience
Anti-mouse/rat CD29 (Integrin beta1)	PE	eBioHMb1-1	12-0291	eBioscience
Anti-mouse CD31	PE	390	102407	BioLegend
Anti-mouse CD34	APC	MEC14.7	119510	BioLegend
Anti-mouse/human CD44	FITC	IM7	11-0441	eBioscience
Anti-mouse CD45	APC	30-F11	103112	BioLegend
Anti-mouse CD73	PE	eBioTY/11.8	12-0731	eBioscience
Anti-mouse CD90.2 (Thy-1.2)	FITC	53-2.1	11-0902	eBioscience
Anti-mouse CD105 (Endoglin)	PE	MJ7/18	12-1051	eBioscience
Anti-mouse PDGFR-α (CD140a)	APC	APA5	135905	BioLegend
Anti-mouse Ly-6A/E (Sca-1)	FITC	D7	11-5981	eBioscience
Rat IgG2a, k Isotype	FITC	eBR2a	11-4321	eBioscience
	PE	eBR2a	12-4321	eBioscience
	APC	eBR2a	17-4321	eBioscience
Rat IgG2b, k Isotype	FITC	eB149/10H5	11-4031	eBioscience
	APC	eB149/10H5	17-4031	eBioscience
Rat IgG1, k Isotype	PE	eBRG1	12-4301	eBioscience
Armenian Hamster IgG Isotype	PE	eBio299Arm	12-4888	eBioscience

Supplementary Table 2. All antibodies used in this study.

Supplementary Fig. 1

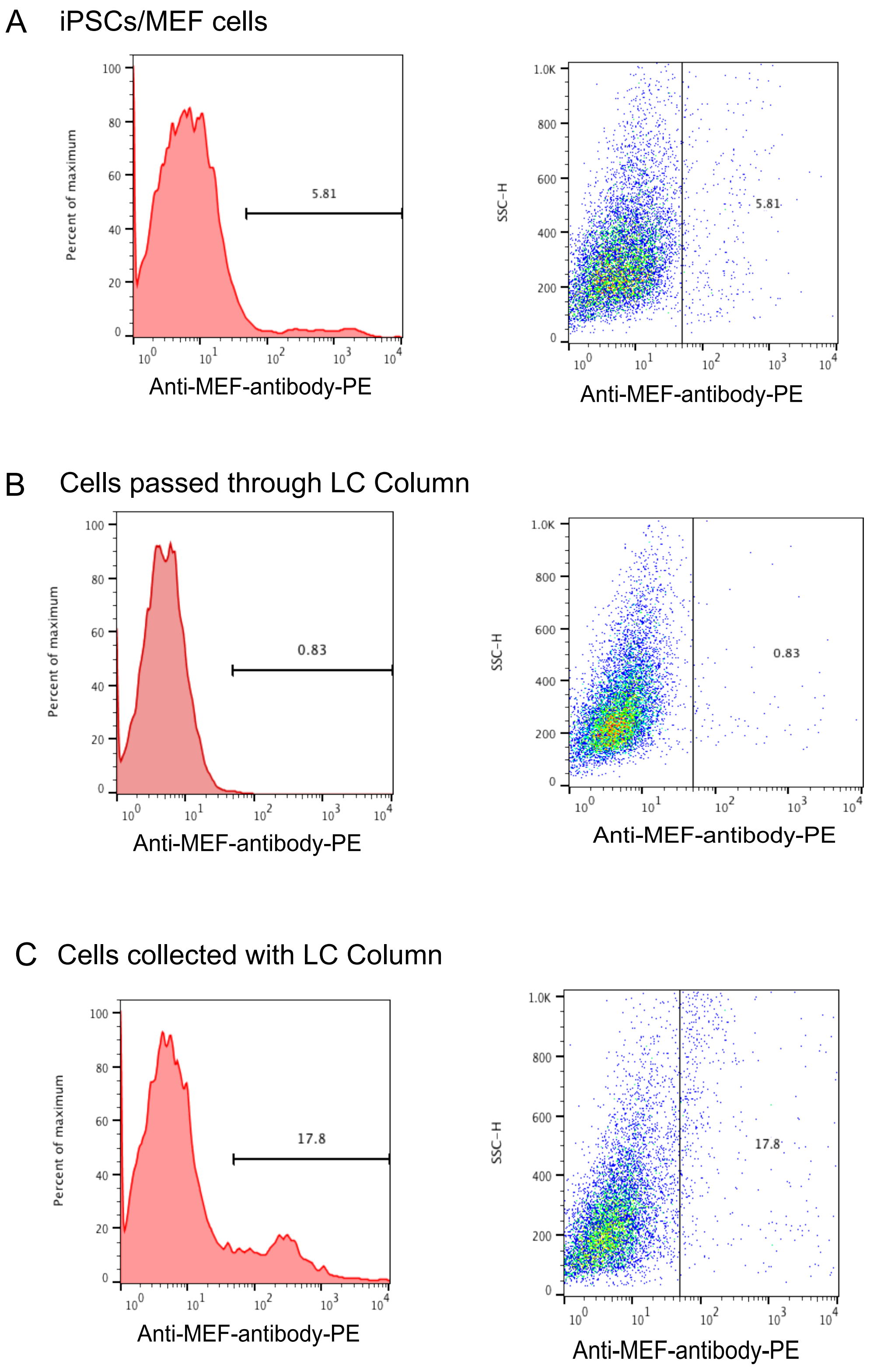

Supplementary Fig. 1. Removal of MEF cells using feeder removal microbeads and LC column. (A) FACS analysis of detached mixture solutions of iPSCs and MEF cells with anti-MEF antibody. (B) The mixture cells after incubation with feeder removal microbeads, were passed through LC column, and passage cells were collected. FACS analysis for passage cells was performed with anti-MEF antibody. (C) Cells attached to LC column were eluted and collected. FACS analysis of elution cells with anti-MEF antibody.

Supplementary Fig. 2

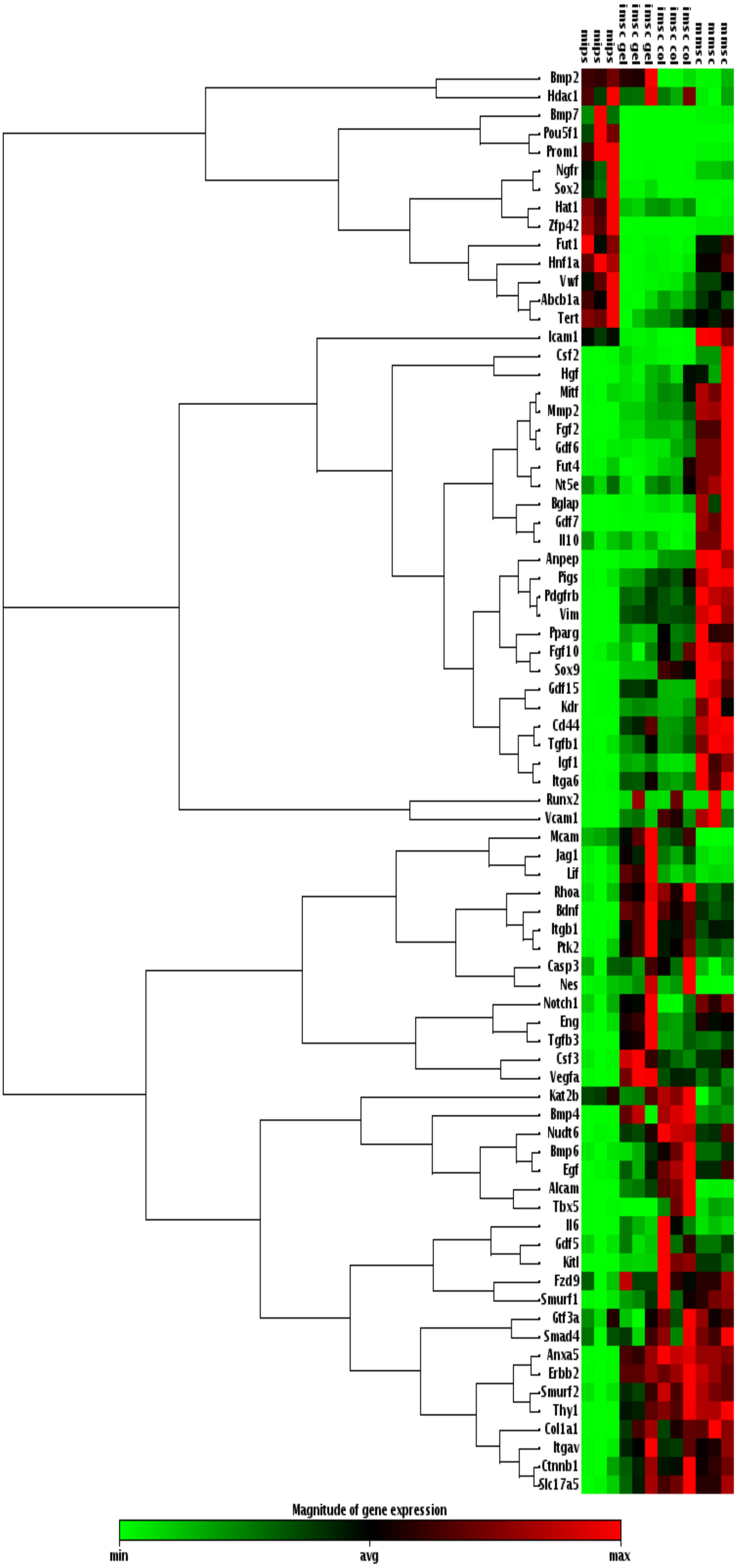

Supplementary Fig. 2. Microarray analysis of iMSCs/G, iMSCs/C, BMMSCs, and iPSCs. A 78 gene expression profile in these cells.

Supplementary Fig.3

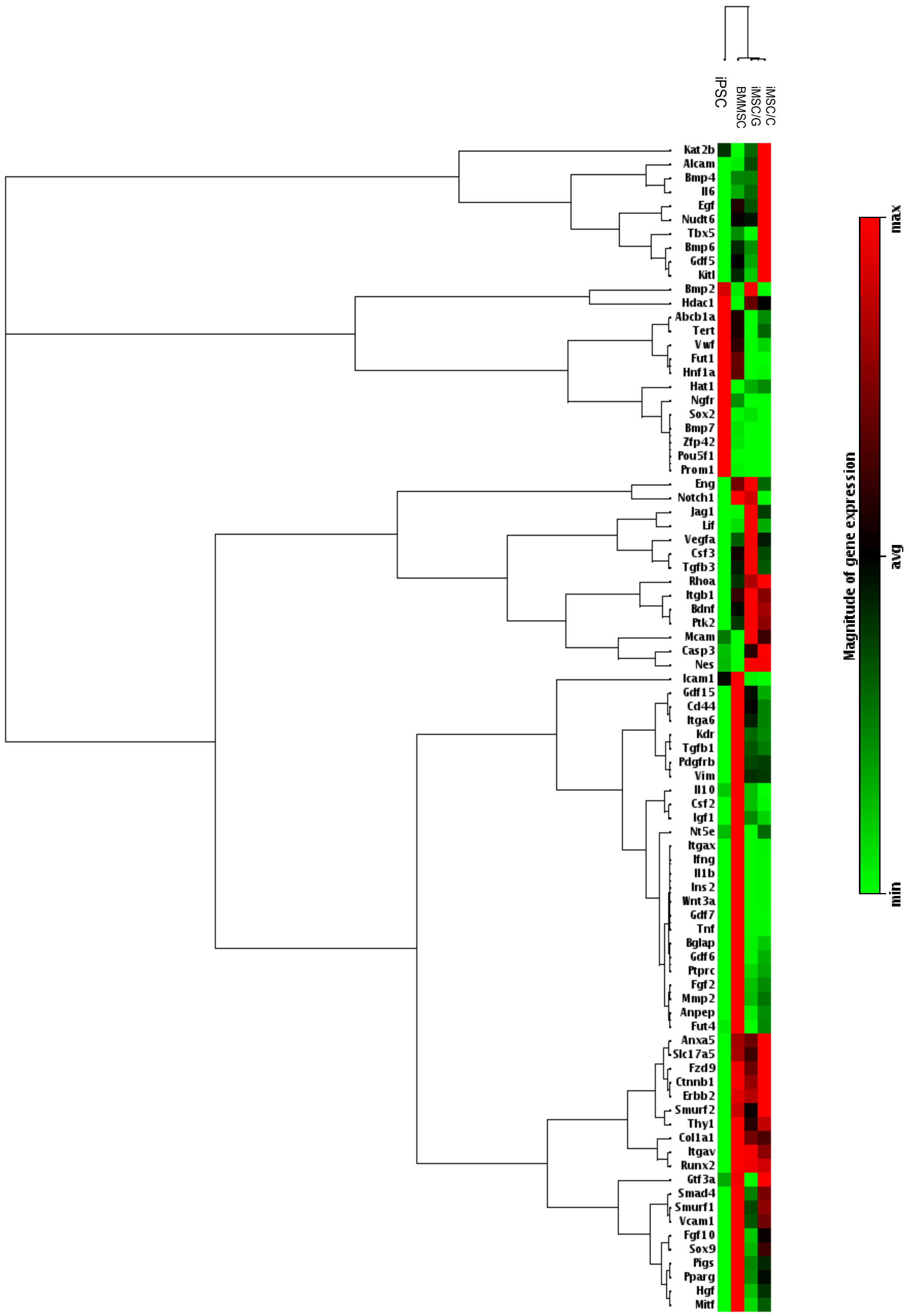

Supplementary Fig. 3. Microarray analysis of iMSCs/G, iMSCs/C, BMMSCs, and iPSCs.
A clustering analysis by selecting 50 genes that were upregulated by 2 fold in BMMSCs relative to iPSCs.
Each column represents average gene expression composed of three samples.

Supplementary Fig.4

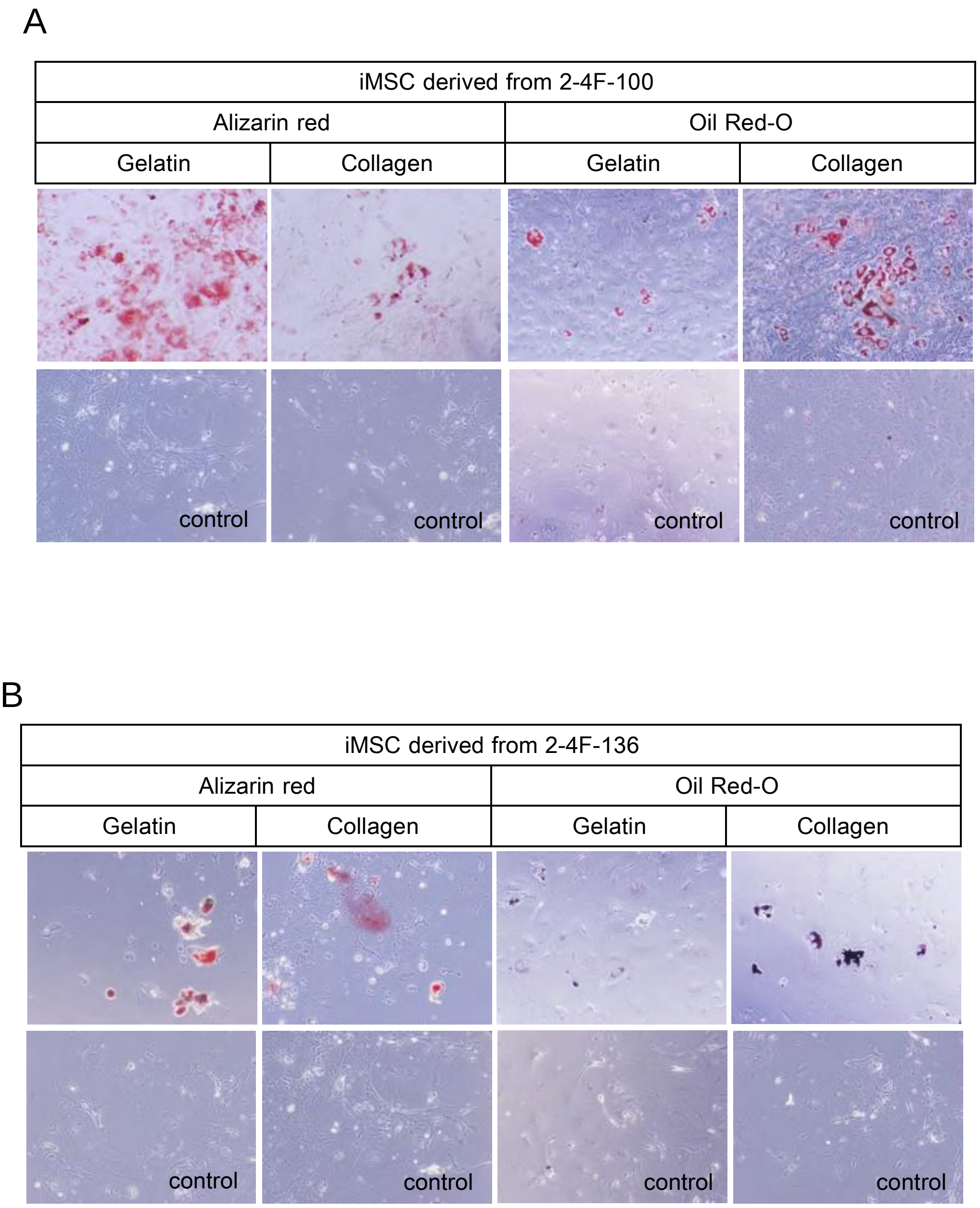

Supplementary Fig. 4. Osteogenic and adipogenic differentiation of P3 iMSC/G and C (2A-4F-100 and 2A-4F-136 lines). (A) P3-iMSCs (2A-4F-100) were cultured in medium containing osteogenic differentiation factors and stained with Alizarin red, and cultured in adipose-differentiation medium and stained with Oil Red-O at 12 days. (B) P3-iMSCs (2A-4F-136) were cultured in osteogenic differentiation factors and stained with Alizarin red, and cultured in adipose-differentiation medium and sataind with Oli Red-O at 12 days.
